# Imaging of Preclinical Endometrial Cancer Models for Monitoring Tumor Progression and Response to Targeted Therapy

**DOI:** 10.3390/cancers11121885

**Published:** 2019-11-27

**Authors:** Heidi Espedal, Tina Fonnes, Kristine E. Fasmer, Camilla Krakstad, Ingfrid S. Haldorsen

**Affiliations:** 1 Department of Clinical Medicine, University of Bergen, 5021 Bergen, Norway; kristine.eldevik.fasmer@helse-bergen.no; 2 Mohn Medical Imaging and Visualization Centre, Department of Radiology, Haukeland University Hospital, 5021 Bergen, Norway; 3 Centre for Cancer Biomarkers, Department of Clinical Science, University of Bergen, 5021 Bergen, Norway; tina.fonnes@uib.no (T.F.); camilla.krakstad@med.uib.no (C.K.); 4 Department of Obstetrics and Gynecology, Haukeland University Hospital, 5021 Bergen, Norway

**Keywords:** preclinical imaging, imaging biomarkers, PDX models, mouse models, gynecological cancer, endometrial cancer, positron emission tomography, magnetic resonance imaging, computed tomography, optical imaging

## Abstract

Endometrial cancer is the most common gynecologic malignancy in industrialized countries. Most patients are cured by surgery; however, about 15% of the patients develop recurrence with limited treatment options. Patient-derived tumor xenograft (PDX) mouse models represent useful tools for preclinical evaluation of new therapies and biomarker identification. Preclinical imaging by magnetic resonance imaging (MRI), positron emission tomography-computed tomography (PET-CT), single-photon emission computed tomography (SPECT) and optical imaging during disease progression enables visualization and quantification of functional tumor characteristics, which may serve as imaging biomarkers guiding targeted therapies. A critical question, however, is whether the in vivo model systems mimic the disease setting in patients to such an extent that the imaging biomarkers may be translatable to the clinic. The primary objective of this review is to give an overview of current and novel preclinical imaging methods relevant for endometrial cancer animal models. Furthermore, we highlight how these advanced imaging methods depict pathogenic mechanisms important for tumor progression that represent potential targets for treatment in endometrial cancer.

## 1. Introduction

Endometrial cancer (EC) is the most common gynecologic malignancy in industrialized countries and the incidence is increasing [1,2]. Most patients present with early-stage disease and are cured by surgery; however, about 15% develop recurrence with limited treatment options and poor survival [3,4]. Although the identification of different genetic alterations has defined specific molecular subtypes with potential for more individualized therapy in EC, the clinical treatment mostly remains the same across molecular subtypes [4,5].

New, promising therapeutics based on preclinical models frequently fail to produce similar effects in clinical trials. This may be related to poor preclinical model systems such as immortalized cell lines implanted subcutaneously in mice, thus lacking metastatic potential and immediate relevance for the human setting. However, during the last decade preclinical cancer models have advanced with the introduction of orthotopic models (tumor cells implanted in the organ of origin) and patient-derived tumor xenograft (PDX) models, both putatively more translatable and transferable to the clinic. Orthotopic PDX models have shown to mimic various human cancers in terms of histopathologic characteristics, genetic- and molecular alterations, metastatic potential and therapeutic response [6]. Animal models for the most common types of gynecologic cancers have been developed, including PDX of endometrial, cervical and ovarian cancer [7,8,9].

Monitoring intraabdominal gynecologic tumor growth is challenging and requires an advanced imaging system. Traditionally, it required sacrificing a large number of animals for various ex vivo assays and endpoint measurement of tumor size. These measurements derived from the same time point (at sacrifice) inherently provide only static tumor information not capturing the dynamically changing and interactive cascade of signaling events that induce tumor progression. Additionally, during therapeutic intervention some molecularly targeted drugs may yield effect without reducing the tumor volume [10] and this effect will consequently not be detected using this approach. Importantly, robust and valid imaging biomarkers for early prediction of treatment response prior to detectable tumor size reduction, may open the avenue for early tailoring of more individualized treatment strategies in cancer.

The use of small-animal imaging in cancer research has increased over the last decade, especially as the development of non-invasive advanced functional imaging techniques has allowed for in vivo longitudinal monitoring of tumor growth, metastatic spread and therapeutic response. Preclinical imaging therefore represents a useful tool for unravelling new imaging biomarkers for prediction and evaluation of treatment response that eventually can be translated into the clinic [11]. Our research group was, to our knowledge, the first to present a multimodal imaging set-up, similar to that routinely employed at primary diagnostic work-up in the clinic, to monitor tumor growth in an orthotopic model of EC [12]. This review will give an overview of available preclinical imaging modalities relevant for gynecological cancers, in particular focusing on EC. Furthermore, we will highlight novel possibilities for refined tumor characterization by emerging new functional imaging techniques and discuss some of the challenges with preclinical imaging in EC.

## 2. Literature Search

The PubMed/Medline and Web of Science databases were searched for articles published up to September 2019 with filtering for animal studies using the following terms and combinations thereof: “endometrial cancer/carcinoma” or “uterine cancer” in combination with “imaging”, “magnetic resonance imaging”, “positron emission tomography”, “single photon emission computed tomography” “computed tomography”, “ultrasound”, “bioluminescence” and “fluorescence”. In addition, we also searched the reference lists of selected studies and authors to identify additional relevant articles. All studies recognized were assessed for relevance by checking the title and abstract. All irrelevant articles, studies without access to the full text of the publication, non-English publications, letters and proceedings were excluded.

## 3. Imaging in Endometrial Cancer

Imaging plays a central role in the management of EC patients. Although EC is formally surgiopathologically staged according to the International Federation of Gynaecology and Obstetrics (FIGO) staging system [13], preoperative imaging findings guide primary surgical treatment and are especially useful to stratify high-risk patients for lymphadenectomy, which is a major clinical challenge [14]. Although guidelines for state-of-the-art preoperative imaging in EC exist [15], differences in access to advanced imaging facilities and dissimilar local preferences lead to variable practices across centers. In humans, transvaginal ultrasound and/or magnetic resonance imaging (MRI) is typically performed preoperatively to assess local tumor extent. Computed tomography (CT) alone or CT combined with positron emission tomography (PET-CT) are useful to assess both abdominal spread to pelvic- and paraaortic lymph nodes as well as distant spread [16]. For EC animal models MRI, PET-CT, CT and single-photon emission computed tomography (SPECT) have been used to depict and monitor tumor growth. Furthermore, these imaging methods enable non-invasive evaluation and quantification of treatment efficacy using novel therapies and depict imaging markers that are closely linked to treatment response. Monitoring of preclinical EC tumor growth using ultrasound could potentially also represent a valuable adjunct to these imaging modalities; however, we are not aware of any preclinical EC studies using this method. Optical imaging using fluorescence can be used to detect sentinel lymph nodes (SLN) in EC with high accuracy [17,18,19,20] and optical imaging using fluorescence and bioluminescence has been widely used as an additional tool in preclinical EC studies.

### 3.1. MRI

MRI is a highly versatile modality with standard sequences yielding high soft-tissue resolution providing detailed information on anatomy, and advanced imaging techniques allow depiction of functional and microstructural properties of the tissue. Diffusion-weighted (DW)-MRI is a functional imaging method depicting random diffusion of water molecules that reflects microstructural tissue characteristics. Typically, malignant tumors exhibit higher cellular density which putatively induces restricted water diffusion; thus, malignant tumors are often distinguished from normal tissue by its restricted diffusion. The DW images using different diffusion weighting (b-values) are used to generate apparent diffusion coefficient (ADC) maps on which the diffusion coefficient of the tissue can be measured. Interestingly, changes in tumor ADC value, with normalization towards non-restricted diffusion during treatment, have been linked to a favorable treatment response [21]. Dynamic contrast-enhanced (DCE)-MRI is a different functional imaging method that depicts microvascular features and yields imaging parameters reflecting tissue perfusion and permeability. For EC diagnosis, conventional pelvic MRI is an excellent method to assess myometrial- and cervical stroma invasion [16], and both DW- and DCE-MRI are considered promising supplementary MR sequences in EC for the prediction of advanced stage and an aggressive clinical phenotype [22,23].

**Figure 1:**
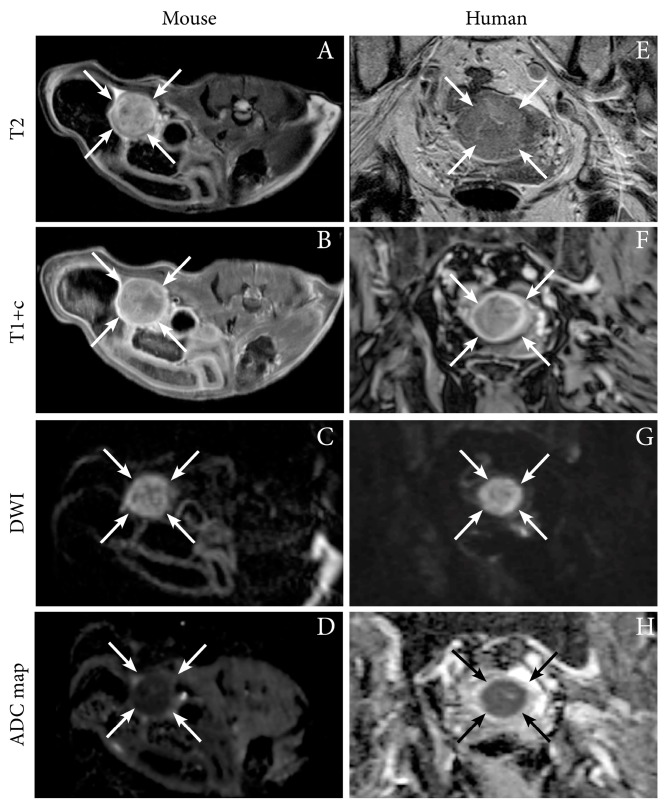
Axial magnetic resonance (MR) images depicting tumor (arrows) in an orthotopic endometrial cancer (EC) mouse model (Ishikawa cells) (**A**–**D**), and corresponding axial MRI images visualizing a uterine tumor (arrows) in an 87-year-old woman with EC (grade 2 endometroid, FIGO stage IIIC1; same patient as in Figure 2) (E–H). (**A,E**) T2-weighted images depict hyperintense tumors and (**B,F**) T1-weighted contrast-enhanced images (T1+c) depict moderately enhancing uterine tumors. (**C,G**) Both the preclinical- and human tumors exhibit restricted diffusion with hyperintensity on high *b*-value diffusion-weighted imaging (DWI) and (**D,H**) corresponding hypointensity on the apparent diffusion coefficient (ADC) maps. Images A–D are reproduced under the open access CC BY license from a previous publication [12].

We have successfully imaged an orthotopic mouse model of EC using MRI, illustrating the feasibility of anatomical MR sequences (T1 and T2-weighted) to depict tumor growth in the uterine horns in mice [12]. Interestingly, the hyperintense tumor signal on T2-weighted series and the hypointensity of the tumor (relative to the surrounding myometrium) on contrast-enhanced (CE) T1-weighted series resemble that observed in human EC (Figure 1). Furthermore, the tumors in mice also exhibit restricted diffusion on DW-MRI with hyperintensity on high b-value images and corresponding low ADC value on the ADC map, being similar to that characteristically observed in humans (Figure 1, Table 1) [12]. For further insight into characteristic imaging findings at conventional imaging and novel promising imaging methods in human EC, we direct the readers to our recent review [16].

The loss of the phosphatase and tensin homolog (PTEN) is a frequent genetic aberration in EC [5], and PTEN loss leads to alterations in the oncogenic PI3K signaling pathway and impaired homologous recombination (HR) repair of DNA breaks [24]. In a treatment study, T2-weighted MRI was used in a genetic mouse model of PTEN-deficient EC to show reduced tumor volume as a result of a synergistic effect to combined treatment with PI3K- (BKM120) and poly (ADP-ribose) polymerase (PARP) inhibitors (Olaparib) (Table 1) [25]. Similarly, T2-weighted MRI was used to show the tumor size dependency on the mammalian target of rapamycin (mTOR) inhibitor rapamycin in a mouse genetic model where EC developed after deletion of the tumor suppressor liver kinase 1B (*Lkb1*), a negative regulator of the AMP-activated protein kinase (AMPK)-mTOR pathway (Table 1) [26].

### 3.2. PET

**Figure 2:**
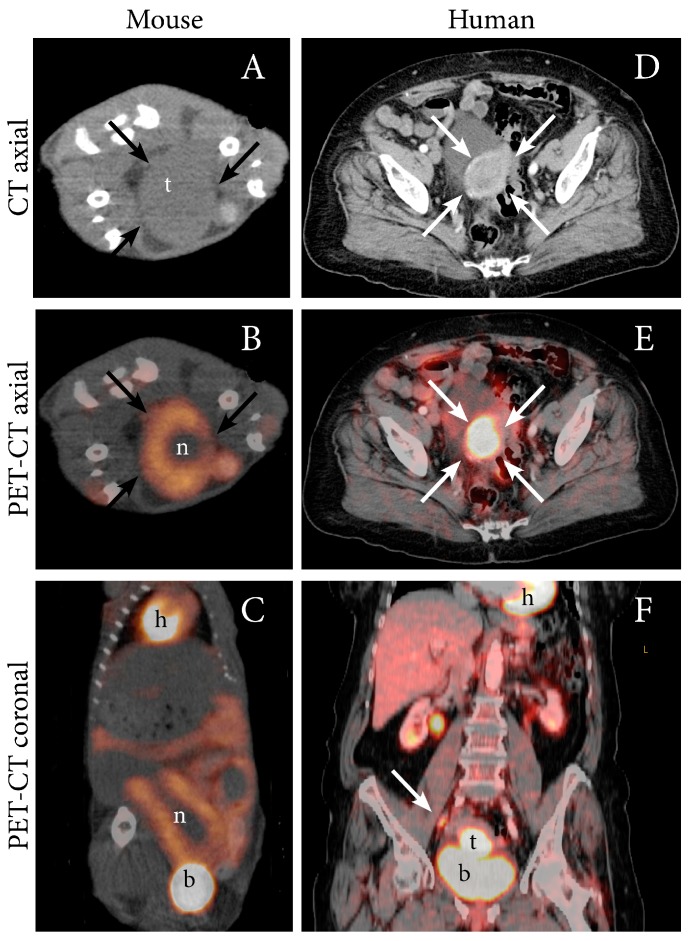
FDG PET-CT depicting an FDG-avid tumor in an orthotopic patient-derived xenograft (PDX) model of EC (grade 3 endometrioid) (**A**–**C**), and in an 87-year-old woman with EC (grade 2 endometroid, FIGO stage IIIC1; same patient as in Figure 1) (**D**–**F**). (**A**) In the mouse model, axial non-contrast CT imaging depicts a large tumor (t) (arrows) in the abdomen whereas (**B**) axial (**C**) and coronal FDG PET-CT display increased FDG uptake in the periphery of the tumor (arrows) and a central necrotic core (n). (**D**) In the patient, diagnostic contrast-enhanced axial CT image depicts a slightly enhancing primary tumor (arrows) and (**E**) axial and (**F**) coronal FDG PET-CT depict an FDG-avid primary uterine tumor (arrow) and a metastatic parailiac lymph node (arrow). Physiologic FDG uptake in the heart (h), liver, kidneys, renal pelvis and intestines and urinary FDG excretion to the bladder (b) is visible.

PET imaging in oncology is most often performed using the ^18^F-labeled glucose, fluorodeoxyglucose (FDG). Malignant cells are metabolically characterized by elevated energy demands and will normally have an increased uptake of glucose. Cancers will therefore typically exhibit high uptake of FDG generating contrast in PET images. PET-CT can detect lymph node metastases in EC with high accuracy, thus preoperative FDG-PET imaging is often recommended in high-risk EC [27,28]. In addition to elevated FDG uptake in metastatic lymph nodes, primary EC are also typically highly FDG avid [27]. Increased FDG uptake in an orthotopic tumor model from both Ishikawa cells and from human endometrioid grade 3 EC (PDX) has been demonstrated by our group using small-animal PET (Table 1) [12], and representative images illustrating the similar PET-CT findings observed in the PDX model and in human EC (endometroid grade 2, FIGO IIIC1) is shown in Figure 2.

In a preclinical EC treatment study, it was recently shown that maximum standardized uptake values (SUVmax) decreased in lung metastases established from EC cell lines KLA and AN3CA by inhibiting the PI3K-pathway using ellagic acid (Table 1) [29].

### 3.3. CT

CT of the thorax, abdomen and pelvis is widely used to detect lymph node metastases and distant spread in EC patients [16]. In preclinical studies, CT has been used to detect both local and advanced disease. In an estrogen-controlled orthotopic model of EC using contrast-enhanced CT (CE-CT), image-derived tumor volume was found to be positively correlated to tumor net weight at necroscopy (Table 1) [7]. CT was also used to detect lung metastases in a genetic mouse model where conditional inactivation of a downstream target of the transforming growth factor β (TGFβ) receptor, activin-like kinase 5 (*Alk5*), led to EC (Table 1) [30]. Furthermore, CT-assessed regression of lung metastases after ovariectomy indicated that the tumors were hormone-dependent [30].

### 3.4. SPECT

SPECT is a nuclear medicine imaging technique based on the detection of gamma rays such as technetium-99 m (^99m^Tc) and iodine-123 (^123^I). Clinically, SPECT-CT with ^99m^Tc nanocolloid/tin colloid has been used preoperatively to identify SLN to stratify for lymphadenectomy in low-risk EC patients [19,31]. In preclinical EC models, SPECT has been used to monitor treatment efficacy of two different strains of oncolytic viruses (Copenhagen and Wyeth vaccinia virus) in subcutaneous AN3CA and ARK-2 cell-line xenografts (Table 1) [32].

### 3.5. Optical Imaging

**Figure 3:**
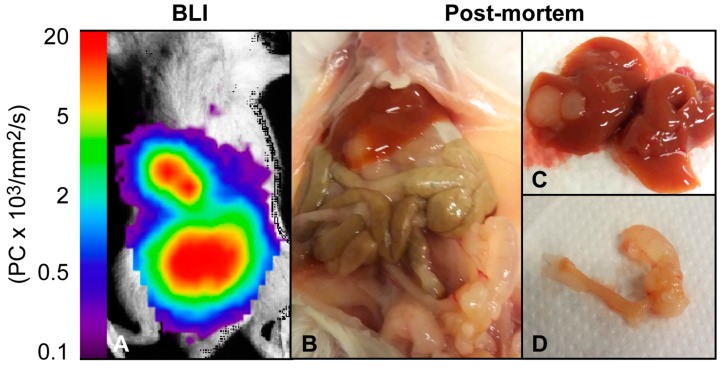
Bioluminescence imaging of an orthotopic EC mouse model. (**A**) Bioluminescence image and (**B**) post-mortem examination of a female NSG mouse ten weeks post orthotopic implantation with luciferase-expressing Hec1B cells. (**D**) Post-mortem examination revealed a primary uterine tumor and (**C**) liver metastases corresponding to the bioluminescence signal.

Optical imaging, which can be performed using bioluminescence (BLI) or fluorescence (FLI) imaging techniques, is an excellent tool for visualization of tumor growth, metastases and treatment effects in preclinical models. BLI requires transfection with luciferase-expressing reporter genes, and as normal cells do not express luciferase, BLI provides excellent sensitivity for detecting tumor cells and high signal-to-noise ratio [33,34]. Unfortunately, this also causes BLI to be ineligible for imaging of PDX models, where genetic alteration of cells is unwarranted. In preclinical EC, BLI imaging has been employed longitudinally to monitor tumor growth and metastatic spread [12,35] and to demonstrate estrogen dependent tumor growth in orthotopic mouse models (Table 1) [7]. A representative BLI image of a mouse orthotopically implanted with luciferase-expressing Hec1b tumor cells is shown together with the corresponding necroscopy finding in Figure 3. In addition to visualizing tumor growth, BLI can be used to monitor the activity of specific signaling pathways, including the identification of significantly reduced activity in the NF-κB pathway following treatment with the heat shock protein inhibitor NVP-AUY922 [36] (Table 1).

Fluorescent imaging (FLI) techniques are increasingly applied in preclinical research. Red fluorescence generated by low doses of 5-aminolevulinic acid was recently demonstrated to allow visualization of EC xenografts following ultrasound microbubble- and polyethyleneimine-mediated knock-down of the enzyme ferrocheletase [37]. Furthermore, FLI using green fluorescent protein has been employed to study the anti-tumor effect of the mTOR inhibitor rapamycin in EC models with different PTEN expression levels. Rapamycin was found to more effectively inhibit tumor growth in PTEN-negative EC tumors compared to PTEN-positive [38]. Similar to SLN-mapping using fluorescent dyes in humans, fluorescence-guided resection of primary tumor and metastatic lymph nodes in an orthotopic rabbit model of EC yielded overall good sensitivity and specificity overall [39].

## 4. Emerging Novel Imaging Techniques Relevant for Preclinical EC Models

Novel imaging methods and new tracers have the potential to yield clinically relevant cancer biomarkers. Monitoring tumor growth in animal models using these imaging methods represents an ideal research platform for testing and validation of cancer biomarkers prior to potential implementation in the clinic. We will highlight promising and novel imaging techniques potentially relevant for preclinical imaging of EC models using examples from breast and gynecologic cancers.

### 4.1. Radioligands for Visualization of Target-Specific Expression in EC

Targeted radioisotopes that bind to specific genetic alterations may identify subgroups of patients that are likely to respond to corresponding targeted therapies, and may therefore serve as an imaging method (using PET and SPECT) for treatment stratification in EC. Isotopes are summarized in Table 2.

Targeting of the human epidermal growth factor receptor-2 (HER2) is implemented in the treatment of HER2-positive breast cancer, but is less studied for treatment of EC. We have recently shown that high HER2 expression is associated with aggressive disease and poor survival in EC [41]. ^89^Zr-labeled pertuzumab (^89^Zr-pertuzumab) has been used to detect HER2-positive breast cancer in small human cohorts [42,43] and to delineate tumors and show treatment effects from trastuzumab-emtansine (T-DM1) in HER2-positive breast cancer xenografts [44]. Similarly, PET-CT with radiolabeled pertuzumab (^64^Cu-NOTA-pertuzumab) was used for non-invasive detection and monitoring of HER2 tumor expression, enabling the detection of primary tumors and peritoneal metastases in both subcutaneous and orthotopic models of ovarian cancer [45].

Cancer antigen 125 (CA125) is an important biomarker in clinical use for ovarian cancer; however, CA125 has also been shown to predict lymph node metastases in EC [46]. A preclinical radiotracer using ^89^Zr labeled mAb-B43.13 (^89^Zr-DFO-B43.13) to target CA125 expression by PET was recently developed and validated in OVCAR3 ovarian xenografts [47], and this approach seems highly relevant for future testing also in preclinical EC models.

It has been shown that tumor expression of epithelial membrane protein 2, EMP2 is associated with aggressive disease in EC, and that EMP2 represents a novel biomarker for EC development [48,49]. Using radiolabeled antibody fragments of EMP2, ^64^Cu-DOTA-EMP2, this tracer was able to detect tumor growth in a cell-line based Hec1a EC xenografts model in mice by PET [50].

The recently discovered G-protein coupled estrogen receptor (GPER) is expressed in tumors where estrogen- and progesterone receptors are downregulated, and is associated with poor survival in EC [51,52]. A ^99m^Tc-labeled GPER ligand for use with SPECT was recently developed and validated in EC Hec50 xenografts [53].

### 4.2. Oncologic PET Tracers Relevant for EC

A wide range of novel PET tracers are currently being developed with the aim of depicting relevant biological processes and molecular targets in oncology, but presently there is little experience with PET tracers beyond FDG in EC. We describe some of the more general radiotracers with potential relevance for patient stratification and treatment assessment in EC. A summary is given in Table 3.

^18^F-labeled thymidine (FLT), is a PET tracer used to depict tissue with actively proliferating cells. FLT is taken up and trapped intracellularly after phosphorylation by thymidine kinase 1 during the S-phase of the cell cycle. Although not yet tested clinically for EC, we have demonstrated the feasibility of FLT-PET to detect and monitor tumor growth in our EC mouse model [12]. For a number of different tumors including animal models of breast- and ovarian cancers, FLT-PET has been used to evaluate treatment response [54,55,56,57,58,59,60].

Unopposed estrogen stimulation is a central oncogenic mechanism driving the development of the majority of ECs (type I; endometrioid) [61]. Furthermore, receptor status in EC provides prognostic information, where loss of estrogen/progesterone receptor (ER/PR) predicts lymph node metastases and poor survival [62]. Thus, PET using ^18^F-fluoroestradiol (FES), to non-invasively depict and quantify whole-tumor expression of estrogen, has long been investigated for the clinical management of EC. Interestingly, Tsujikawa et al. found that the FDG-to-FES SUV ratio is related to tumor aggressiveness and that the more aggressive tumors depend less on estrogen and more on glucose metabolism. Furthermore, tumor FES-PET avidity correlated well with ERα expression [63,64]. In a preclinical study, FES-PET was used to detect early treatment response to the endocrine therapy fulvestrant in an ER+ breast cancer xenograft model (MCF7), a response which was not detectable by FDG-PET [65]. Similarly, in ER+ breast xenografts (ZR-75-1) FES-PET could detect treatment response to fulvestrant, whereas FDG- and ^18^F-fluoromisonidazole (FMISO; hypoxia tracer)-PET did not [66].

In general, hypoxia leads to a reduced response to chemo- and radiotherapy, and we have shown that imaging and tissue markers for hypoxia predict reduced survival in EC [67]. Therefore, detecting hypoxic tumors by preoperative functional imaging might have therapeutic consequences and improve risk-stratification. Several PET tracers for hypoxia have been developed and FMISO and ^18^F-fluoroazomycin-arabinofuranoside (FAZA) represent two of the most common commercially available hypoxia tracers. Neither FAZA nor FMISO have been tested in preclinical or clinical EC, but both have shown promise for detection of hypoxic regions in cervical cancer [68,69] and in animal models of cervical and ovarian cancer [57,70,71].

### 4.3. Advanced MRI Sequences Relevant for EC

Advanced functional MRI sequences can be used to evaluate therapeutic response in cancer [21]. For preclinical gynecologic cancer models, but not yet for EC, DW- and DCE-MRI have been used to assess therapeutic cellular and vascular response following targeted therapies. DW-MRI was used to demonstrate treatment effects from perifosine, a PI3K-inhibitor, and the chemotherapeutic drug cisplatin in a genetic model of ovarian cancer [72]. Furthermore, DW- and DCE-MRI was used for the non-invasive monitoring of treatment response during administration of the dual PI3K/mTOR-inhibitor BEZ235 in two different subcutaneous ovarian cell-line xenograft models [73]. Rofstad and colleagues have applied DCE-MRI to characterize hypoxia in the tumor microenvironment finding that low values of the contrast agent transfer constant (K^trans^) is associated with areas of hypoxia, resistance to radiotherapy and higher metastatic potential in cervical cancer models [74,75,76].

### 4.4. Advanced Image Analyses

One of the main advantages of the PET technology is the possibility to perform absolute quantification – also in small animals. Clinically, PET images are usually acquired by conventional static scanning, typically one hour post-injection for FDG-PET, and analyses are based on the semi-quantitative and variation-prone parameter SUV [77]. In contrast, dynamic PET depicts the distribution of the tracer in space and time from the time of injection, thus reflecting both the early uptake- and late distribution phase and the metabolism. Pharmacokinetic modeling from dynamic imaging can potentially yield functional tumor information beyond that represented by SUV and better characterize tumor heterogeneity and monitor therapeutic response [78,79]. Interestingly, dynamic FDG-PET using compartment modeling demonstrated treatment response from chemotherapeutics (doxorubicin, paclitaxel and carboplatin) in three different breast cancer xenografts in mice, a response which was not detectable using conventional SUV parameters [79].

## 5. Imaging-Related Challenges in Preclinical Endometrial Cancer Models 

Primary gynecologic cancers are localized in the small pelvis in close proximity to the bladder and the bowels; this often makes it challenging to distinguish tumor from surrounding, normal tissues. As many PET tracers are excreted through the urinary track to the bladder; spill-over from the bladder due to the partial volume effect (PVE) can affect the delineation of tracer avid tumor tissue adjacent to the bladder and sometimes mask the entire tumor, especially in orthotopic mouse models. Furthermore, physiologic FDG uptake in intestines and other abdominal organs can affect the tumor segmentation even when fasting the mice prior to imaging (to reduce intestinal FDG uptake). Similarly, FLT physiological uptake in proliferating tissues e.g., the intestines [12] and FMISO uptake by anaerobic fecal bacteria [80] can complicate tumor segmentation.

For absolute quantification of dynamic PET or DCE-MRI images, the arterial input function (AIF) is needed. To acquire AIF, the gold standard requires manual blood sampling at several time-points after tracer/contrast agent injection. As the blood volume in small animals is very small (estimated 55–70 mL/kg body weight [81]), the method is technically challenging, subject to considerable variation and does not allow for longitudinal studies. Several alternatives to derive the AIF exists, including a pump-driven arteriovenous shunt connected to a coincidence counter (e.g., Twilite by Swisstrace), a microfluidic chip blood-counting system [82] or indirect estimates based on the dynamic images (image-derived input function, IDIF) or using a population-based input function (with or without a blood sample for scaling). Which method that yields the most accurate quantification is however, debated. Furthermore, the technical ease of the imaging procedure and the accumulated burden for the animals undergoing sequential examinations are critical, if these practical interventional methods are to be feasible for a broader use.

## 6. Conclusions

Detection and monitoring of tumor growth in preclinical EC models by using clinical imaging methods such as CT, MRI, SPECT and PET is feasible. These dedicated small-animal imaging technologies enable the identification of imaging biomarkers reflecting functional, microstructural and metabolic tumor features relevant for EC phenotype. The majority of preclinical studies exploring new targeted therapies with potential for personalizing EC treatment, still rely on endpoint volumetric measurements of tumor size. The therapeutic effect of molecularly targeted drugs not having an immediate effect on tumor size may consequently be overlooked. However, novel functional imaging techniques and new tracers enable quantification and depiction of molecularly targetable pathogenic mechanisms, yielding information beyond what has previously been obtainable. However, the development of relevant target-specific imaging biomarkers in EC is still in its infancy, and new imaging techniques and tracers are likely to be developed in the near future. Furthermore, all promising imaging techniques will require careful validation and clinical testing prior to potential translation into the clinic. Importantly, imaging biomarkers identified by preclinical models with subsequent validation in clinical patient series, are likely to play a central role in future developments of new targeted treatment strategies in gynecologic cancer.

## Figures and Tables

**Table 1:** Preclinical imaging of endometrial cancer.

Imaging Modality/ Sequence	Study Purpose	Imaging Characteristics	Animal Model	Ref
**MRI** T1, T1+C, T2, DW, ADC	Present preclinical imaging findings using multiple imaging techniques	Tumor can be delineated using anatomic sequences and exhibits restricted diffusion with low ADC-values	Ishikawa cells, orthotopic, NSG mice	[12]
T2	Explore therapeutic effect of combined PI3K (BKM120) and PARP-inhibitor (Olaparib) treatment	Tumor volume decreased after combined treatment (synergistic effect)	Genetic mouse model (*PTEN*/*Lkb1*- deficient [40])	[25]
T2	Present a novel genetic mouse model	Tumor volume dependent on rapamycin treatment (mTOR inhibitor).	Genetic mouse model (*Lkb1*-deficient)	[26]
**CT** (CE-CT)	Present an estrogen-controllable mouse model with image-guided monitoring of tumor-growth	Correlation between CT-assessed tumor volume and tumor weight at necroscopy	Ishikawa cells, orthotopic, estrogen-controllable, athymic nude mice	[7]
CT	Present a novel genetic mouse model	Detection of lung metastases and regression of metastases post-therapy (ovariectomy)	Genetic mouse model, hormone dependent (*Alk5-*deficient)	[30]
**PET** FDG	Present preclinical imaging findings using multiple techniques	Growth of primary tumor and metastases can be detected Total lesion glycolysis was calculated (SUVmean x MTV)	Ishikawa cells and PDX-model, orthotopic, NSG mice	[12]
FDG	Explore effect of PI3K-inhibtor (ellagic acid)	Decreased SUVmax in metastases (lungs) after treatment	Cell lines KLE and AN3CA injected iv. in BALB/C nude mice	[29]
**SPECT** (NIS reporter gene)	Explore effect of oncolytic therapy	Tumor volume decreased after therapy	Cell lines AN3CA and ARK-2, subcutaneous athymic mice	[32]
**Optical** BLI	Generation and characterization of a mouse model	BLI signal increases over time. Metastatic growth detected	Hec1A cells, orthotopic, athymic nude mice	[35]
	Present preclinical imaging findings using multiple techniques	BLI signal increases over time. Metastatic growth detected	Ishikawa cells, orthotopic, NSG mice	[12]
	Present an estrogen-controllable mouse model with image-guided monitoring of tumor-growth	BLI signal increases in estrogen-treated mice	Ishikawa cells, orthotopic, athymic nude mice	[7]
	Evaluate effect of Hsp90-inhibtor (NVP-AUY922)	NVP-AUY922 treatment reduces activity in the NF-κB pathway detected by BLI signal	Ishikawa cells, subcutaneous, (unknown mice strain	[36]
FLI	Optimization of fluorescent signal	Low dose ALA permits detection of tumor by FLI following knockdown of FECH by ultrasound microbubbles and polyethyleneimine	Hec1a cells, subcutaneous, BALB/c- nude mice	[37]
	Investigate mTOR treatment in tumors of different PTEN-status (+/-)	Decreased GFP signal in PTEN- compared to PTEN+ for rapamycin-treated tumors	Hec1a (PTEN+) and Ishikawa (PTEN-) subcutaneous, BALB/c-nude mice	[38]
	Explore fluorescence-guided resection of tumor and metastases	Detection and surgical removal of fluorescent tumor tissue with high sensitivity and specificity	VX2 rabbit tumor cells, orthotopic, White New Zealand rabbits	[39]

**Abbreviations**: ADC apparent diffusion coefficient, ALA 5-aminolevulinic acid, Alk5 activin-like kinase 5, BLI bioluminescent imaging, CE contrast-enhanced, CT computed tomography, DW diffusion-weighted, FDG fluorodeoxyglucose, FECH ferrocheletase, FLI fluorescence imaging, GFP green fluorescent protein, Hsp90 heat shock protein 90, iv. intravenous, Lkb1 liver kinase b1, MRI magnetic resonance imaging, mTOR mammalian target of rapamycin, MTV metabolic tumor volume, NF-κB nuclear factor kappa-light-chain-enhancer of activated B cells, NSG NOD scid gamma mouse, PARP poly (ADP-ribose) polymerase, PDX patient-derived xenograft, PET positron emission tomography, PTEN phosphatase and tensin homolog, SPECT single-photon emission computed tomography, SUV standardized uptake value.

**Table 2:** Target-specific radiotracers relevant for endometrial cancer.

Target/Modality	Clinical Relevance/Finding	Tracer	Preclinical Animal Model/Finding
HER2–PET	HER2 positivity predicts aggressive disease and poor outcome [41].	^89^Zr-pertuzumab	Uptake in human HER2+ breast cancer [42,43] and mouse HER2+ xenografts (BT-474) including evaluation of tumor size change after treatment with HER2-targeted antibody-drug conjugate (T-DM1) [44].
		^64^Cu-NOTA-pertuzumab	High specificity to HER2 expression and delineation of tumor and metastases in orthotopic and subcutaneous ovarian cancer xenografts [45].
EMP2–PET	High EMP2 expression predicts aggressive disease [48,49].	^64^Cu-DOTA-EMP2	High uptake and delineation of subcutaneous tumors of EMP2-overexpressing Hec1a-cells [50].
CA125–PET	High serum CA125 predicts lymph node metastases [46].	^89^Zr-DFO-mAb-B43.13	Delineation of subcutaneous ovarian cancer xenografts (OVCAR3)[47].
GPER–SPECT	High GPER expression is associated with poor survival [51,52].	^99m^Tc-GPER	Uptake in subcutaneous EC (Hec50) and breast cancer (MCF7/HER2–18) xenografts [53].

**Abbreviations:** CA125 Cancer antigen 125, DFO desferrioxamine (chelating agent), DOTA 1,4,7,10-tetraazacyclododecane-N,N′,N′,N′″-tetraacetic acid (chelating agent), EMP2 epithelial membrane protein-2, HER2 human epidermal growth factor-2, GPER G-protein coupled estrogen receptor, mAb monoclonal antibody, NOTA 1,4,7-triazacyclononane-triacetic acid (chelating agent), T-DM1 trastuzumab-emtansine.

**Table 3:** Novel imaging techniques relevant for clinical and preclinical imaging in endometrial cancer.

Target	Imaging Modality/Sequence	Clinical Relevance	Clinical Findings	Preclinical Application and Findings
Tumor proliferation	FLT-PET	Sustained proliferation is a hallmark of cancer, including EC.	No human studies performed in EC.	Growth of primary tumor and metastases can be detected and monitored longitudinally in EC mouse models [12].
				FLT can detect treatment response in breast- and ovarian cancer models [54,55,56,57,58,59,60].
Estrogen status	FES-PET	Estrogen drives development of type 1/endometrioid EC, receptor status can predict survival [61,62].	FES-FDG ratio can predict grade in EC, FES-PET avidity is linked to ERα expression [63,64].	Shown to predict early treatment response to fulvestrant in ER+ breast cancer xenografts [65,66].
Tumor hypoxia	FMISO-PET FAZA-PET DCE-MRI (K^trans^)	Hypoxia predicts poor survival in EC [67].	FMISO- and FAZA-PET depict hypoxic regions in cervical cancer [68,69].	FMISO- and FAZA-PET depict growth of subcutaneous ovarian xenografts and enable monitoring of treatment response (chemotherapy) [57]. Low tumor values of K^trans^ is associated with hypoxia in cervical cancer models [74,75,76].
Tumor heterogeneity and vascularity	DW- and DCE MRI	DW- and DCE-MRI are valuable supplements to conventional diagnostic MRI sequences [22,23].	DCE-parameters (F_b_, K^trans^ and V_e_) are lower in tumor than normal myometrium, tumor ADC is negatively correlated to tumor volume [23].	DWI (ADC value is negatively correlated to Ki67 proliferation index) to assess treatment response by PI3K-inhibitor perifosine and cisplatin in ovarian xenografts [72]. DWI (↑ADC value) and DCE (↑V_e_ ) to demonstrate BEZ235 (dual PI3K/mTOR inhibitor) treatment response in ovarian xenografts. [73].
	Pharmaco- kinetic modeling, dynamic PET		More accurate quantification and better characterization of tumor heterogeneity in breast cancer [78].	Rate constants K_1_ and K_2_ (perfusion) was higher and K_3_ was lower (metabolism) in breast cancer xenografts treated with chemotherapy; this response was not detectable by traditional SUV analyses [79].

**Abbreviations:** ADC apparent diffusion coefficient, DCE dynamic-contrast enhanced, DW diffusion-weighted, ERα estrogen receptor α, FAZA fluoroazomycin-arabinofuranoside, FDG fluorodeoxyglucose, FLT fluorothymidine, FMISO fluoromisonidazole, K^trans^ volume transfer constant, MRI magnetic resonance imaging, mTOR mammalian target of rapamycin, PET positron emission tomography, PI3K phosphoinositide 3-kinase, SUV standardized uptake value, V_e_ extravascular extracellular space.
